# The Meaning and Factors That Influence the Concept of Body Image: Systematic Review and Meta-Ethnography from the Perspectives of Adolescents

**DOI:** 10.3390/ijerph18031140

**Published:** 2021-01-28

**Authors:** Glòria Tort-Nasarre, Mercè Pollina Pocallet, Eva Artigues-Barberà

**Affiliations:** 1 Department of Nursing, Faculty of Nursing and Physiotherapy, University of Lleida, Carrer de Montserrat Roig, 225198 Lleida, Spain; eartigues.lleida.ics@gencat.cat; 2 Health Education Research Group (GREpS), Faculty of Nursing and Physiotherapy, University of Lleida, Carrer de Montserrat Roig, 225198 Lleida, Spain; 3 Calaf Primary Care Center, Cta. Llarga, 19.08280 Calaf. Barcelona, Gerència Territorial Catalunya Central, Catalan Health Institute (ICS), 08272 St. Fruitós del Bages, Spain; 4 Bellpuig Primary Care Center, Diputació, 5. 25250 Bellpuig, Lleida, Gerència Territorial Lleida, Catalan Health Institute (ICS), Rambla Ferran, 44, 25007 Lleida, Spain; mpollina.lleida.ics@gencat.cat; 5 Catalan Health Institute (ICS), Primary Care Lleida, Rambla Ferran, 44, 25007 Lleida, Spain; 6 Research Support Unit Lleida, Fundació Institut Universitari per a la Recerca a l’Atenció Primària de Salut Jordi Gol i Gurina (IDIAPJGol) Rambla Ferran, 44, 25007 Lleida, Spain; 7 Research Group in Therapies in Primary Care (GRETAPS), Rambla Ferran, 44, 25007 Lleida, Spain

**Keywords:** adolescents, body image, dissatisfaction, distortion, self-esteem, self-image, systematic review, meta-ethnography

## Abstract

Community care nurses educate adolescents about body image, but their interventions appear to be ineffective. Body dissatisfaction predicts unhealthy behaviors among adolescents. This study aimed to understand the meanings and factors that influence the concept of body image through a systematic review and meta-ethnography of qualitative studies from the perspective of adolescents. Ten studies published from 2009–2019 were identified by a search of relevant systematic databases between September and December 2019. The review followed the seven steps of meta-ethnography developed by Noblit and Hare, including a line-of-argument. The synthesis revealed six themes: self-perception of body image; opinions of friends and colleagues; opinions of family; specific features of the school environment; expectations perceived across the mass-media; and strategies, practices, and self-management of body image. An explanatory model was developed that showed adolescents’ development of body image and the path towards its establishment. In conclusion, these results should be considered to implement strategies to promote a healthy body image in adolescents by community health and mental health nurses.

## 1. Introduction

Body image (BI) is the internal representation of one′s external appearance [1] and encompasses self-perceptions related to the body and personal attitudes, including thoughts, beliefs, feelings, and behaviors [2]. It is a multidimensional representation [3] made up of four components: global subjective satisfaction (evaluation of the body); affection (feelings associated with the body); cognitions (investment in appearance and beliefs about the body); and behaviors (avoidance of situations of body exposure) [1]. Therefore, BI is a complex phenomenon that includes many components related to gender, ethnic and sociocultural factors [2,4]. Effective measures regarding BI must address individual feelings, including satisfaction/dissatisfaction; physical self-awareness; beliefs; ideas; and behaviors regarding appearance [4].

Body dissatisfaction is defined as negative thoughts and feelings of a person about his/her body. It is related to negative evaluations of body size, shape, and weight; this generally implies a perceived discrepancy between the evaluation of one’s body and the ideal body [1,5]. The prevalence of body dissatisfaction in adolescents is varied in different western countries; according to the results of the cross-sectional HBSC (Health Behavior in School-aged Children) 2013–2014 study of European and American adolescents, at the age of 15, 40% of girls and 22% of boys are dissatisfied with their body weight [6].

Dissatisfaction with BI during the early stages of adolescence has been related to poorer self-esteem and anticipates different issues: depressive symptoms; higher body mass index; less physical activity; clinical eating disorders, poorer dietary quality, and disordered eating (i.e., fashionable dietary behaviors and the use of food for emotional regulation); and behaviors aimed at losing weight (i.e., diets, food restrictions, and modification of eating habits) that, at the same time, could lead to distortion of one’s own BI [7,8]. Thus, some authors associate certain internal factors of BI with: body dissatisfaction; concepts of the body [9], perceptions of beauty and ideals of appearance transmitted by society and media [10,11]; attitude towards the self and towards others [9]; and gender differences and perspectives [12]. Likewise, other authors identify the media together with family and friends as external factors that influence BI [7,9,12]. Education, tutors and material status may also be factors associated with determining BI assessment [13,14,15,16,17]

Independent BI interventions have been multiple and heterogeneous [18,19]. Several studies show that small effects in improving BI are achieved by using techniques such as: discussing the role of cognition in BI; restructuring cognition; changing negative body language; teaching monitoring; using guided imagery, exposure exercises, and size estimation exercises; and training in stress management and in prevention of relapse, e.g., using behavior change techniques [19]. Finally, to be effective, health promotion strategies must be focused on BI based on the voice of adolescents, their experiences, and their social and cultural framework. From here, we considered it important to conduct a review of qualitative studies on the meaning of BI in adolescents from western countries. The results of our research must guide strategies for promoting positive BI, and preventing dissatisfaction about BI, in the community setting. We addressed several questions: What are the central themes addressed by qualitative studies on the meaning of BI in adolescents from western countries? What factors influencing BI are explored? Where does qualitative research on BI and dissatisfaction during adolescence bring us?

## 2. Aim

The objective of this study was to analyze the meaning of, and factors influencing, the body image construct, from the perspectives of adolescents in western countries, through an interpretative systematic review of qualitative studies.

## 3. Methods

### 3.1. Design

We performed a systematic review and an interpretative synthesis, following the meta-ethnography model proposed by Noblit and Hare [20].

### 3.2. Search Strategy

We carried out our research between September and December 2019. We previously determined the inclusion and exclusion criteria (Table 1).

The inclusion criteria for the sample selection required that the original studies described the meaning, attribution, and experiences of BI in healthy adolescents. Moreover, data from these primary studies had to be obtained from the perspective of adolescents. The original reports must have used a qualitative approach in relation to data collection and data analysis. Studies using mixed methods were eligible for inclusion if it was possible to extract the results derived from qualitative research.

We preliminarily identified studies through searching relevant electronic databases, by using Medical Subject Headings (MeSH) terms and text words.

Table 2 describes the final search strategy, which was adapted to the selected databases according to the specific language used in each one.

We searched the Web of Science, PubMed, PsycINFO, CINAHL, Scopus, and the Cochrane Register. We set the publication date for the search from 2009 to 2019 and considered only peer-reviewed studies published in English. Finally, we complemented the process by searching for key authors. Using the last strategy we found several studies, but they were not incorporated as they did not meet the inclusion criteria and were not exactly focused on the subject of the review.

### 3.3. Selection and Summary of the Studies

**Figure 1:**
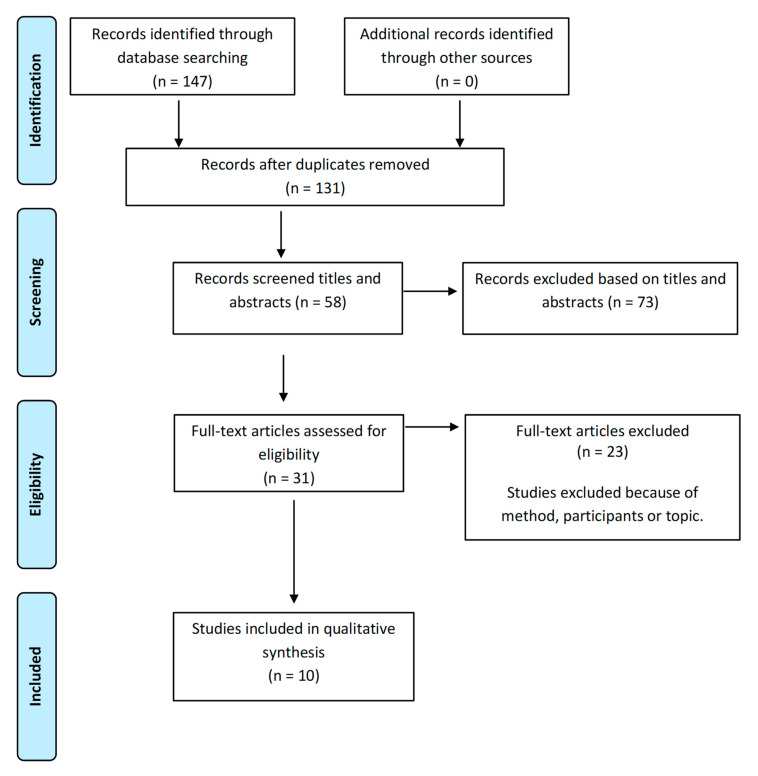
PRISMA Flow-Diagram of Screening Process for Review.

The main investigator (GTN) performed the systematic literature search and was responsible for reviewing the *n* = 147 potential studies according to title, abstract and full text. Studies were excluded when they did not meet the inclusion criteria, did not focus sufficiently on the topic, were from specific ethnic groups, or data had not been collected from an adolescent perspective. Disagreements were resolved by discussion and by reference to the full article by another investigator (EAB). Finally, the research team (GTN, MPP, EAB) agreed on the studies (*n* = 10) that should be included in the synthesis. The flowchart is displayed in Figure 1.

### 3.4. Quality Assessment

The ten included papers were assessed for quality by the review team independently using the Critical Appraisal Skills Programme (CASP) tool [21]. None were excluded according to quality criteria.

This research did not require approval, since all the studies included in the review had already been approved by their respective ethics committee.

### 3.5. Synthesis

The ten studies were synthesized using Nobit and Hare′s [20] seven-stage method. In the first phase, we identified the topic of interest provided by the qualitative studies. The second phase involved selecting the studies to include in the synthesis. This phase generated a repeated process of reading the studies to identify a list of metaphors in each of them and the affiliation of disparate and common themes. We drew a map of the units of meaning, codes, and themes of the phenomenon under study. We used the ATLAS-TI vs.7 software (Development GmbH. Berlin. Germany) for this phase. The topics in each article were initially identified by the principal investigator (GTN), and later discussed and analyzed by the entire research team (GTN, MPP, EAB). Disagreements were resolved by rereading from top to bottom all selected articles. Once we identified the main concepts in each article, we made a search for the presence of these concepts in the rest of the documents. During this process, we ensured that each key concept took on similar meanings in all documents. Moreover, we identified those that were unique or specific to one or more studies.

We started the synthesis with the first published article [22], then worked through the studies in chronological order of publication. We performed the comparison process by identifying the themes in the first study, and then adding others as they emerged (Table 3).

Subsequently, we developed a “line of argument” [20]. In this phase, it was possible to reorganize the results generating a new interpretation of the phenomenon explained by the data. In this way, we achieved a synthesis that was not only the sum of the individual parts of each article, but also provided a meticulous interpretation, and preserved the integrity of each study. This phase was carried out by GTN and then discussed by the full group of researchers. Finally, we obtained an explanatory model of the construct, the meaning, and the factors that contribute to developing BI in adolescence in a western context.

## 4. Results

### 4.1. Characteristics of Included Studies

We summarize the main characteristics of the 10 articles included in the synthesis in Table 4.

All the manuscripts are from scientific journals and published in the English language. The studies were carried out in six countries: three in Ireland [22,26,28], two in Sweden [24,31], two in the USA [9,30], one in India [23], one in Norway [27], and another in the UK [29]. Eight of these studies use qualitative designs [22,23,24,25,26,27,28,29], and the other two use mixed methods [9,30]. Likewise, all perform thematic content analysis. As data collection method, five studies apply focus groups [22,26,27,28,29], three use semi-structured interviews [23,24,25] and two employ focus group and individual survey [9,30].

### 4.2. Description of Themes

**Figure 2:**
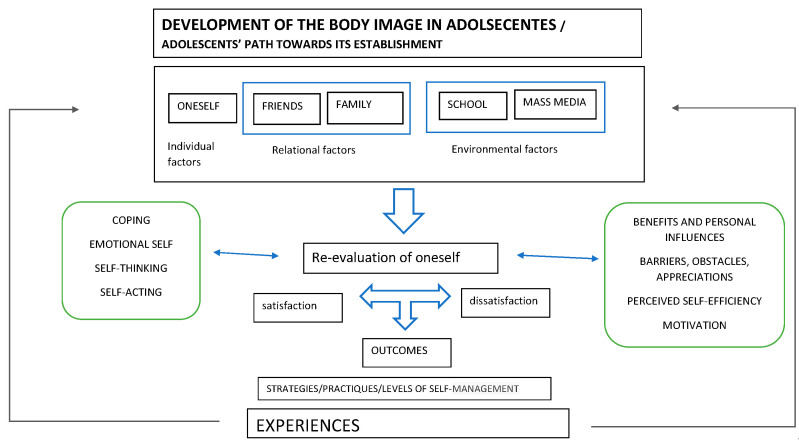
Line-of-argument: explanatory model.

We extracted six themes that influence the process of developing BI, according to the opinion of adolescents: (1) self-perception of BI; (2) opinions of friends and colleagues; (3) opinions of family and parents; (4) specific features of the school environment that influence BI; (5) perceived expectations of BI across the mass-media; and (6) strategies, practices, and self-management for BI. These themes are closely related to each other. Figure 2 illustrates the line-of-argument synthesis that demonstrates the key processes in understanding adolescents′ meaning of BI.

#### 4.2.1. Self-Perception of BI

Six of the studies provide self-perception data that influence the desire for an ideal BI [9,22,23,26,27,31]. Adolescents explain how self-esteem, self-confidence, insecurity, acceptance, self-protection, and anxiety are part of their internal dialogue and beliefs about their BI. Some young people express concern about physical appearance, such as being too fat, gaining weight, aspiring to be prettier, putting on makeup to cover acne [22,23]. Therefore, they express a desire for a different BI. They show negative emotions, such as anger, sadness, guilt or frustration [9,22,23]. Some are aware that they must stop focusing on their body to have a healthier self-esteem. Self-confidence is crucial [24,26], as well as appreciating themselves as they are [22]. Satisfaction with BI is related to acceptance [24,27]. In two articles [9,25], adolescents gave more importance to interior happiness, intelligence, and kindness, than to physical appearance.

#### 4.2.2. Relevance of the Opinions of Friends and Colleagues on BI

Eight studies examine the influence of peers on the ideal of BI [9,22,23,24,26,27,28,29]. Participants express thoughts and emotions about the pressure of being accepted; the fear of being rejected, excluded, or judged; and the feeling of being watched, compared, or criticized for their physical appearance [9,22,27,28,29]. Others claim that they do not compare themselves to friends and aspire to the ideal of BI that they define [23,24]. Body weight is a point of constant vigilance and criticism, jokes, teasing, and exclusion among peers. Such situations can last over time and affect mental health by producing low self-esteem, eating disorders, self-harm, and even suicide [22,28]. The motivation to be accepted drives adolescents to follow fashion trends, and change their appearance by dieting, fasting, and practicing physical exercise [26]. Consequently, adolescents copy standard models and talk about losing weight, dieting, wearing clothes that make them more attractive, and practicing sports.

#### 4.2.3. Relevance of the Opinions of Family and Parents on BI

Six studies provide data related to the influence of parents on BI in terms of dissatisfaction and satisfaction [9,23,24,26,29,30]. Several adolescents comment on the importance of pressure derived from the family, specifying that it influences then via the way they dictate diet and how they model eating habits and self-confidence [29]. It was sometimes shown that mothers persuade their daughters to copy the styles and fashion imposed by celebrities [26]. Adolescents think that their parents are concerned about their health: they encourage them to a healthy, non-sedentary lifestyle and healthy eating [23]. Other studies show that parents express opinions about clothes and haircuts, and judge them according to their own ideals [24]. Other parents pressure their children to lose weight and conform to standard models [9]. Among the actions undertaken by adolescents because of the messages received by parents, we identify factors that facilitate and predispose the appearance of unhealthy behaviors or reinforce pre-existing ones.

#### 4.2.4. Relevance of Specific Features of the School Environment That Influence the BI

Only two of the selected articles provide results on how the school and teachers influence adolescents′ beliefs about BI [29,30]. In Brunette et al. [30] some adolescents express how the school environment encourages them to learn strategies that help to mitigate the harmful aspects of negative comparison between equals, including those received through the mass media. They also mention the benefit of voluntarily participating in a school group dealing with BI. In the study of Sharpe et al. [29] some participants point to teachers as a valuable source of support and give some recommendations.

#### 4.2.5. Perceived Expectations on BI across the Mass Media

Seven studies provide data on the relationship between mass-media and the perception of satisfaction or dissatisfaction with BI [9,22,23,25,26,27,30]. Some participants comment that they are influenced by the image of certain international actresses or celebrities [22,26,27]. Some adolescents, especially girls, copy and imitate fashion, clothing, and hairstyles of celebrities to feel more comfortable with their individual appearance [23,26]. There are also mixed opinions on the role of the media in developing dissatisfaction with the weight of adolescent women [23]. Some girls convey that they are aware that companies use appearance and BI for commercial purposes [25].On an emotional level, the images spread by the mass media make them feel frustrated if they do not achieve the ideals [9]. Other teens instead feel uncomfortable with the messages and decided not to look at them [25]. Some participants comment that selfies are an important part of their life. They add that they need to receive likes to feel comfortable with their BI and increase their level of self-esteem [30].

#### 4.2.6. Strategies, Practices, and Self-Management for the Ideal BI: Diet and Physical Exercise

Seven of the studies refer to the use of diet and exercise as self-management practices related to physical appearance [22,23,24,26,27,28,29]. Diet and exercise are used as strategies for weight loss [24,26], sometimes including fad diets [22]. However, some people are not conscious of such diets [23] and inadvertently engage themselves in unhealthy behaviors [26]. The goal is to become equals [28,29], mostly through diet for females and bodybuilding for males [26,27,28]. Therefore, they pretend to look like celebrities [26] and attract male attention [22,29].

Positive peer influences are manifested by congratulations, sources of encouragement, and opinions to motivate dieting, physical activity, and exercise and to counteract useless pressures [28,29]. In the same way, support and motivation by mothers represent positive influences [23]. Sports stars and dancers influence healthy behaviors [26,27]. However, some adolescents already conceive physical fitness and caring for the body as a part of health care, turning exercise into a natural and important part of life, generating joy, fun, and friendship [24]. One of the studies [29] propose different prevention strategies with early interventions on body dissatisfaction and diet. The effectiveness of the last strategy is shown in the study by Brunette et al. [30], along with self-acceptance and acceptance of differences, trust, and diversity.

### 4.3. Explanatory Model

The explanatory model derived from these results shows that the BI elaboration process is a dynamic, multidimensional, and complex phenomenon. It responds to internal and external stimuli experienced by the adolescent himself, and not simply social pressure, friendship, and school environment. According to this model, the reevaluation of oneself is a crucial element that influences the meaning of, and satisfaction or dissatisfaction with, BI. Beliefs and emotions reinforce an evaluation of oneself that leads one to accept or reject bodily self-image. As a result of this reevaluation, some of the adolescents diet and exercise as a self-management strategy to achieve an ideal weight or image that matches the canons of beauty. However, the resistance to accept one’s body does not necessarily imply unhealthy diets or disproportionate physical exercise as strategies for weight control. Individual coping is crucial for addressing one′s image and adolescent identity. We show that there are barriers and obstacles to, and also positive appreciations for, the achievement of a healthy BI in adolescence. However, there are adolescents with an efficient perceived self-efficacy and with positive appreciations about their body, even when it does not fit into the beauty canons established in Western society. All these factors should be considered in prevention programs, and promotion of a healthy BI.

## 5. Discussion

This synthesis suggests that the BI construct in adolescents has multiple meanings and that it does not necessarily imply dissatisfaction when there is a mismatch between actual and desired BI. BI is established in a much more complex way than simply through the messages received by the mass media and the pressure from friends. The family and the school environment play a decisive role as protective factors when applicable, providing tools to promote self-esteem, security, and confidence. We identified six themes from the analysis of the various studies.

1. Self-perception of BI defines the way of thinking and feeling about oneself in relation to body image. Messages about the body can be interpreted in a distorted way [32]. Therefore addressing misperceptions about weight is not enough for the prevention and promotion of a healthy body image in adolescents [33,34]. The studies analyzed show that the problems of distortion of body image facilitate behaviors aimed at losing weight. Dissatisfaction with body image during the early stages of adolescence has been related to poorer self-esteem [7], more in adolescent girls than in boys [35].
2. Relevance of the opinions of friends and colleagues on BI relates to the messages that adolescents receive from their friends. However, Willis et al. [36] provide results that contradict the common perception that overweight or obesity in adolescents is related to body dissatisfaction. Some of the overweight teens do not support the perception of friends or family about BI.
3. Relevance of the opinions of family and parents on BI. Both the negative influence of friends and family members are considered barriers to a healthy BI; however, they can be facilitators when messages are received positively by the adolescent [37]. Adolescents with high levels of body dissatisfaction may also experience higher levels of depression and less positive social interactions that lead to a decrease in family connection [38]. Consequently, considering the point of view of each adolescent, it is essential to include the role of parents in health educational interventions to develop a healthy BI.
4. Relevance of specific features of the school environment that influence BI. There are few studies which investigate the influence of school and teachers on the development of BI. Several studies show the benefits of including programs to promote a healthy BI in schools [39], thus evidencing the important role that school plays as a primary context of socialization [40].
5. Perceived expectations of BI across the mass-media. Exposure of physical appearance in social networks is related to different factors: (1) dissatisfaction with weight; (2) drive for thinness and ideal internalization; (3) self-objectification, especially in adolescent girls [10,41]; and (4) increased concerns and beliefs related to appearance [42]. Some of them are aware of the manipulation of the media and are critical of the pressure exerted about obtaining the ideal BI [43]. Nevertheless, the influence of media on adolescent girls should also be considered. Consequently, media literacy is considered necessary [25], as well as the development of prevention strategies. The latter include promoting skills to use the internet and mass media and training adolescents to develop a healthy BI.
6. Strategies, practices, and self-management for the ideal BI. The results of the synthesis agree with the study of Pollina et al. [44] in which it is shown that body dissatisfaction predicts unhealthy behaviors among boys and girls, with different patterns between gender. This dissatisfaction also generates problems of distortion of BI. Moreover, it facilitates behaviors aimed at losing weight (i.e., diets, food restrictions, and modification of eating habits), which are patterns of eating behavior that predict obesity in the future [45].

The present synthesis also yields an explanatory model on the meaning of the BI construct and the process of its elaboration. This model can promote approaches focused on health, well-being, and social justice by emphasizing contextual factors, and not exclusively focusing on weight, BMI, and individual responsibility, as pointed out by Tylka [46]. Obtaining the desired and satisfactory BI can prevent risk behaviors related to (1) dietary habits; (2) exercise; (3) medication; (4) situations of bullying and exclusion; (5) emotional discomfort around self-esteem, self-confidence, and self-acceptance; and (6) mental health diseases, such as distortion of BI, anxiety and depression, which can last into adulthood and old age. In conclusion, in our study, we are create the basis to address a major problem in public health. The results of the present synthesis also reinforce the proposals [47,48] for exploring positive BI in adolescents more deeply.

## 6. Strengths and Limitations of the Review

The strength of the review is reflected in the systematic identification of articles, using a broad and specific search strategy and multiple databases. Furthermore, this rigor is improved by applying the CASP score.

One possible limitation is due to the nature of the methodology, which is related to the use of different qualitative designs. In this review, however, we place the focus on the substantive area provided in each study.

Future research is necessary to determine the extent to which the present results could be generalized in other social and cultural contexts. Indeed, most of the studies reviewed are from western countries or western models of societies in which family, friends, and social factors play the same role.

## 7. Implications for Practice

A model has been developed that can help health professionals and educators to reorient the focus of interventions and programs devoted to this issue.

We should make an effort to create intervention tools to provide adolescents with a distanced, critical, and reflective point of view on the inputs they receive.

To prevent problems and promote public health by providing adolescents with information, instruments, and strategies would help empower adolescents to form the most positive perspective on BI and motivate them to engage in healthy behaviors for to maximize their satisfaction with their BIs. The results reported here may also help investigators formulate intervention strategies for improving BI among pre-adolescent children.

## 8. Conclusions

The present review shows the construct, the meaning, and the key factors that influence the development of BI, according to the perspective of adolescents. The synthesis presents strategies that have a negative impact on the health of adolescents, and factors that are a protective framework for a healthy BI.

The results provide relevant information for nursing professionals who should consider the design and implementation of educational programs on BI and health promotion in childhood and adolescence. These interventions should consider multiple levels, the perspectives of adolescents and the specific needs of each subgroup: girls, boys, peers, friends, etc. In addition, our synthesis identifies protective factors for a healthy BI, such as positive school environment and teacher support, as well as the need for strategies to deal with the impact of mass media messages. These are necessary to understand the needs of adolescents and develop individualized care plans in the earlier stages of life.

## Figures and Tables

**Table 1:** Eligibility criteria.

Inclusion Criteria	Exclusion Criteria
- Studies published in English	- Quantitative studies
- Primary studies/ original research Qualitative studies	- Studies on clinical populations: e.g., people with eating disorders, malformations, and mental illnesses
- Adolescents between 12 and 19 years old	- Studies on pregnant adolescents
- Healthy population	- Studies on high-level adolescent athletes

**Table 2:** Database search strategy.

1. Self esteem	9. Dissatisfaction
2. Self-image	10. Distortion
3. BI	11. 9 or 10
4. 1 or 2 or 3	12. 4, 8 and 11
5. Adolescent *	13. Qualitative research
6. Teenager	14. Qualitative studies
7. Young	15. 13 or 14
8. 5 or 6 or 7	16. 12 and 15

**Table 3:** Quotations from participants and authors to illustrate each theme.

Themes/Categories	Quotations from Participants Primary Studies	Interpretations of Findings Offered by Authors
**Individual Factors:** **Self-Perception of Body Image**		
Emotional self	When I eat something fattening I feel bad. I feel guilty that I am already fat and after eating this, I will become fat [23].	Feeling guilty about myself
	I think I look pretty good. I′m not like very good-looking, but quite normal. It′s not that I′m ugly [24].	Self-confidence
	I get sad when I get chubby [9].	Feeling sad
	People degrade them, make them feel bad about them and that′s where they get more stressed and then they eat those foods [9].	Get stressed
	Everybody should be able to look the way they want to, and there should not be any things that make them feel that they need to change [25].	Be yourself
	Hayley: Some people it could happen to and others it would never. Roisin: Like if the person is confident. Researcher: OK, so you think that helps them not be affected by it? Roisi4: Yeah ′cos if they are really shy and all they would probably be but if they were confident they wouldn′t [26].	Be confident
Thinking self	If you like yourself and you look in the mirror and like what you see in there, you′re confident with people [22].	Self-confidence
	My weight gain is the main concern. I like the way my face is [23].	My concern
	I think there are greater differences between boys than there are between girls. I think most girls want the same things, they want to be slim and Yeah, like, be good looking and slim. But I think the boys here are more into beefing up than the west-end boys are. That′s what I think [27].	As I want to be
	It depends more on talking a lot, being clever and being knowledgeable. Then you can talk about status. I don′t think it′s about appearance. It′s more about being visible at our meetings. This goes for both boys and girls [27].	Important values
	I think most people, at least girls, have an inner wish to be thin and would love to have a body like Britney Spears. This is the goal, but then I think it varies between groups [27].	Wish to be thin
**Relational Factors:** **Relevance of the Opinions of Friends and Colleagues of Body Image**		
Peer surveillance	People our age are very critical of other people and how they look. Like they′re very cruel and they don′t think they′ve got feelings and they make snap judgements about girls and give them low self-esteem [22].	To be overly critical of appearance
	They put pressure on you to get the body they think is right, like soccer players, skinny strong and muscley [28]. Because you get judged. Like everybody judges you, wherever you walk really (…) I think just looking attractive so that people don′t talk about you and say like, ‘Oh she’s fat, she′s ugly, her hair looks ridiculous [29].	Pressure for an ideal body
Peer acceptance	Guys don′t look at me. Nothing about me has changed but the weight gain. I feel that they are not looking at me because of the weight gain….Guys don′t look at me the way they used to [23].	Without acceptance
	Some people in my class they are big and they get bullied a lot, but I stop that bullying [9].	Get bullied
	They like could be fat and everybody′s mocking them so they like exercise because exercise makes you thin [26].	Fear of being mocked
	They [my friends] think that I have strange toes. They usually sit and look at them at gymnastics at school. But I think they do so mostly just to tease me, just for fun. (How does it make you feel?) I don’t care. (Doesn′t it bother you at all?) No [24].	Negative comments are not important
**Relational Factors:** **Relevance of the Opinions of Family and Parents of Body Image**		
Influence of parental opinion	My parents kept telling me, “Go to the gym.” My mom said, “You are leading a sedentary lifestyle”. (Mom) is concerned about my health. She says, “You eat unhealthy food, you keep sitting around [23].	Concerned about my health.
	My mom gets magazines and we would look through them. if we like it [celebrity look] I′d do something like and copy (them) [26]. My mom is mad at me because I (weigh) less than her [9].	Mother pressure
	[We] might talk about that I had my hair cut and [mother says] Oh, that haircut really suits you (...) or if I buy a new top she usually says ‘well, that’s nice′ [24].	Opinions about clothes
	So if you see your parents going on Atkins Diet or something then you′re going to be thinking ‘oh should I be having carbs? Like is it not good to have carbs?’ So then you just think it′s normal to just cut things out of your diet [29].	Pressure from family
Opinion on parental control	My parents, like, tried to make a lot of restrictions and, like, I don′t listen to them anymore [30].	Parental restrictions on social media
	It like gives me anxiety whenever my parents are like ‘okay, I′m gonna just like check something’ and they like actually check my computer history a lot and so like I start to like freak out like even though I′ve done like nothing wrong which makes them like get a message that like I did something even though I′ve done nothing. And so I’m like constantly worried about what I use [30].	Parental vigilance on social media
**Environmental factors:** **Relevance of Specific Features of the School Environment of Body Image**		
Opinion on teachers	If you go to a teacher, they′re going to give you the ‘well it′s wrong and you know what can happen’ [29].	Teacher support
	At this school, they teach us how to like yourself and self-confidence and stuff. This is a really inclusive school and it teaches you how you shouldn′t care how other people think of how you look but in other public schools… Our teachers in health class, like all the umm teachers help us with confidence [and] teach us to build a really thick wall so that this stuff does not get to you and I guess you are more immune to it because you know you are fine and so does your class, too [30].	Positive school environment
**Environmental Factors:** **Perceived Expectations Across the Mass-Media of Body Image**		
Celebrities	A lot of the boys talk about what celebrities they like and then the girls would look up that celebrity and how they look, what they wear and everything and might copy them [26]. You would want to be as skinny as them [22]. If I’m following a female celebrity. they do all these, like, photo shoots and stuff and they look really pretty. So, sometimes, I guess that makes me not feel good? [30] Then everybody started to aspire for it. You become conscious that you have to be a size zero. Earlier you were size 1.Now you are relatively fatter [23].	Copying fashion trends from celebrities
Mas media	Accepting compliments is so much easier than being like [30].	Pressure to get likes
	I feel pretty confident in myself. I mean, I might. look at somebody and be like, ‘Yeah, she′s really pretty’ but. I′m happy with who I am, like I wouldn’t want to be anybody else [30].	Confident in myself
	Like I know sometimes I′ll look at pictures and it′ll make me feel like not happy with myself cause sometimes I′ll wanna look like them [30].	Comparison/unhappy with myself
	Buy this top and it will make you look this thin [25].	Coping fashion trends
**Outcomes: Strategies, Practices, and Self-Management**		
Acting self: diet	Both self-devised diets and prescribed fad diets would be executed, normally for short periods of time [22].	Dieting and prescribed fad diets
	They say something like ‘I′m so fat, I need to get on a diet!’ or something like that, and then people who are actually like more overweight might think like ‘oh they′re saying that and they′re skinnier than me, which means that I′m even worse than them’ [29].	Dieting
	I tend to stick to whole wheat and I don′t have white rice and white bread. I stay away from sugar, cheese. I don′t like oily things. I think it has become a habit because I have stuck to this for quite a while. I never deny myself [23].	Healthy diet
	You know the vinegar, she drinks that and it kind of stops her from eating and it actually just makes her stomach feel full [26].	Dieting and prescribed fad diets
Acting self: sport	My brother, he′s only 15, his team the under 17 s, they are, they have to start working in the gym now and they have to be on this diet, and my brother′s friend plays for Munster and they have to be on this proper diet like they can’t eat chocolate bars [26].	Sport practice
	Like if you were kind of fat they wouldn′t really like talk to you. They′d leave you out of all the games and say picking teams for a match you′d always be the last picked just because you′re fat and you weren′t sporty [28].	Not to be sporty
	Exercise as a natural part of life. Exercise as joyful and health-promoting [24].	Healthy exercise

**Table 4:** Characteristics of included studies in the review.

N°	Author YEAR País	Aim	Sample Sampling Method	Methodology Data Analysis Data Collection	Emergent Themes	Main Results
1	2017 USA [30]	To explore relations between social media use and body image in early adolescent girls	*n* = 38 girls Age 12–14 years Purposive sampling	Mixed-methods approach using Sociodemographic dates. Mass media usage survey. Six focus group Semi-structured Interview for 50 min. After focus group individual surveys were completed.	Five Themes emerged: - Social media norms - Selfies - Social comparison - Appearance concerns - Social media strategies	- Girls have concerns about appearance and social comparison - Strategies that seemed useful to mitigate the possible negative association between exposure to social media and body image. - Positive parental influence and a supportive school environment.
2	2011 India [23]	To identify the factors recognized as contributing to weight dissatisfaction in Indian adolescent girls and young women	*n* = 10 girls Age 15–21 years Purposive Sampling	A structured interview during 60 to 90 min.	Six themes emerged: - Concerns about appearance - Role of the family - Role of peers - Media pressure - Coping and distress - Desired supports	- Messages from parents, peers and the media creating pressure to lose weight and belief that reducing weight would lead to better life opportunities and greater acceptance from others. - Support from mental health professionals was not desired. - Support from family and friends was considered important.
3	2010 Sweden [24]	To investigate positive body image during adolescence.	*n* = 30 15 boys and 15 girls Age 10–13 years Purposive Sampling	Semi-structured Interview for 60 min.	Four themes emerged: - Satisfaction with own appearance - Views of exercise - Influence from family - Influence from friends	- Adolescents′ satisfaction with their own appearance was characterized by a functional view of the body and an acceptance of bodily imperfections - Negative comments were not given any importance.
4	2018 USA [9]	To determine which characteristics of a American Indian and African American children prefer and to gather their opinions on body image.	*n* = 51 25 girls, 26 boys Age 8–13 years Ethnic group Purposive Sampling	Mixed-methods approach using body image assessment instruments. Eight focus group separated by age and ethnicity. Semi structured questions lasted 75 min.	Two themes emerged: - External factors influencing body image: family, media. - Attitude towards body size: attitudes towards self, attitudes towards others.	- Influence from their parents and the media. - Negative consequences including disordered eating habits, depression, and bullying.
5	2012 Sweden [25]	To examine the topic of appearance ideals from the perspective of 14-year-old adolescents with a positive body image.	*n* = 30 15 girls and 15 boys Age 14 years	Individually Semi-structured interview was used. Lasting 1 h.	Two themes emerged: - Criticism of appearance ideals - A different way of thinking about beauty and attractiveness	- The adolescents with a positive body image were very critical of current ideals, describing them as unnatural and unrealistic, and criticizing media - Defined beauty widely and flexibly, stressed thB importance of looking like ‘oneself’, and conveyed the idea of personality as outplaying looks.
6	2017 Ireland [28]	To investigate how peers influence adolescent body image and whether this influence was positive and/or negative from young peoples′ perspectives	*n* = 111 59 females and 52 males Age 13–18 years Purposive Sampling	17 focus group with single-gender groups.	Seven themes emerged: - Peer modeling - Pressure to conform - Peer surveillance - Failure to conform - Health consequences - Positive peer influences - Age and gender differences	- Peers have an overwhelming negative impact on adolescent body image and, consequently, health. - Peer environment is characterized by a significant pressure to conform to appearance expectations. - Positive peer influences were also revealed but to a far lesser extent.
7	2016 Ireland [26]	To explore common perceptions and influential processes occurring within current Irish appearance culture.	*n* = 39 20 girls and 19 boys Age 12–14 years Purposive Sampling	Eight Focus Groups, four groups of each sex. A semi-structured interview guide. Lasting 30–40 min.	Three themes emerged: - Proximal appearance environment - Distal appearance environment - Personal attributes	- The appearance-related values and behaviors of significant others were highly influential, especially those of peers. - Cultural norms were evident in the young adolescents′ conceptions of the ideal body, and these appeared to be further negotiated and reinforced in proximal contexts.
8	2013 Norway [27]	To investigate how body ideals are discussed and conceptualized among groups of Norwegian youth.	*n* = 63 48 girls and 15 boys Age 16–20 years	Nine focus group. A semi-structured interview guide. Lasting approximately 50–60 min.	Three themes emerged: - Perceptions of Differences between Youth Groups. - Body Talk on How Body Ideals Affect Others - Body Talk Resisting the Cultural Ideal	- Even though the ideals were more or less the same across the groups, the ways of addressing, conceptualizing and discussing body ideals clearly differed between the groups. - Body talk within peer groups seems to contribute when defining adequate levels of effort that should be put into reaching the dominating body ideal
9	2009 Ireland [22]	To investigate the opinions of female adolescents living in Ireland on issues relating to body image and dietary practice	*n* = 124 girls Age 15–16 years Purposive Sampling	16 focus group sessions lasted approximately 40 min. A semi-structured interview guide.	Two themes emerged: - Factors that influence body image - Diet	- The influence of media celebrities was significant. - Peers influenced body dissatisfaction and dieting practices. The results are of concern as the adolescent females were utilizing unhealthy methods of weight control. - A slim body image was deemed important for peer acceptance.
10	2013 UK [29]	To explore adolescents′ views on causes of body dissatisfaction and dieting and recommendations for prevention.	*n* = 22 girls Age 13–15 years Purposive Sampling	4 Focus Groups lasted 1 h. A semi-structured interview guide.	Four themes emerged: - Peer acceptance - Social comparison online - Pressure from family - Pressure from the media and fashion industries.	- Seven areas of recommendation for prevention: building sources of support; learning to be critical of the media; monitoring the school gym; working with parents; educating about signs and symptoms of eating disorders; working with people who have suffered from eating dis-orders; and providing help from professionals.

## Data Availability

The research team has the data under their control. The data will be available to anyone who requests it if the demand is reasonable.
